# Degenerative Cervical Myelopathy induces sex-specific dysbiosis in mice

**DOI:** 10.3389/fmicb.2023.1229783

**Published:** 2023-10-20

**Authors:** Carlos Farkas, Eduardo Retamal-Fredes, Ariel Ávila, Michael G. Fehlings, Pia M. Vidal

**Affiliations:** ^1^Biomedical Science Research Laboratory, Department of Basic Sciences, Faculty of Medicine, Universidad Católica de la Santísima Concepción, Concepción, Chile; ^2^Biomedical Science Research Laboratory, Developmental Neurobiology Unit, Department of Basic Sciences, Faculty of Medicine, Universidad Católica de la Santísima Concepción, Concepción, Chile; ^3^Department of Genetics and Development, Krembil Research Institute, University Health Network, Toronto, ON, Canada; ^4^Spinal Program, Toronto Western Hospital, University Health Network, Toronto, ON, Canada; ^5^Biomedical Science Research Laboratory, Neuroimmunology and Regeneration of the Central Nervous System Unit, Department of Basic Sciences, Faculty of Medicine, Universidad Católica de la Santísima Concepción, Concepción, Chile

**Keywords:** Degenerative Cervical Myelopathy, gut dysbiosis, butyrate, short chain fatty acids, neuroinflammation

## Abstract

Degenerative Cervical Myelopathy (DCM) is the most common cause of spinal cord impairment in elderly populations. It describes a spectrum of disorders that cause progressive spinal cord compression, neurological impairment, loss of bladder and bowel functions, and gastrointestinal dysfunction. The gut microbiota has been recognized as an environmental factor that can modulate both the function of the central nervous system and the immune response through the microbiota-gut-brain axis. Changes in gut microbiota composition or microbiota-producing factors have been linked to the progression and development of several pathologies. However, little is known about the potential role of the gut microbiota in the pathobiology of DCM. Here, DCM was induced in C57BL/6 mice by implanting an aromatic polyether material underneath the C5-6 laminae. The extent of DCM-induced changes in microbiota composition was assessed by 16S rRNA sequencing of the fecal samples. The immune cell composition was assessed using flow cytometry. To date, several bacterial members have been identified using BLAST against the largest collection of metagenome-derived genomes from the mouse gut. In both, female and males DCM caused gut dysbiosis compared to the sham group. However, dysbiosis was more pronounced in males than in females, and several bacterial members of the families Lachnospiraceae and Muribaculaceae were significantly altered in the DCM group. These changes were also associated with altered microbe-derived metabolic changes in propionate-, butyrate-, and lactate-producing bacterial members. Our results demonstrate that DCM causes dynamic changes over time in the gut microbiota, reducing the abundance of butyrate-producing bacteria, and lactate-producing bacteria to a lesser extent. Genome-scale metabolic modeling using gapseq successfully identified pyruvate-to-butanoate and pyruvate-to-propionate reactions involving genes such as Buk and ACH1, respectively. These results provide a better understanding of the sex-specific molecular effects of changes in the gut microbiota on DCM pathobiology.

## Introduction

Traumatic spinal cord injury (SCI) causes loss of descending control over sympathetic preganglionic neurons, leading to dysfunction of the autonomic reflex circuitry, gastrointestinal (GI) tract, and SCI-immune depression syndrome (Brommer et al., 2016; Ueno et al., 2016; Holmes and Blanke, 2019; Ulndreaj et al., 2020). Non-traumatic spinal cord injuries, share some of these features, such as dysfunction of the GI tract (Nouri et al., 2020), increased local neuroinflammation within the spinal cord (Yu et al., 2011; Vidal et al., 2017; Laliberte et al., 2021), and major changes in T cells and monocytes at the systemic level (Ulndreaj et al., 2022). Among the non-traumatic forms of SCI, Degenerative Cervical Myelopathy (DCM) is the most common cause of spinal cord impairment in adults (50 years or older) (Bartels, 2021; Smith et al., 2022). It is caused by chronic compression of the spinal cord due to normal aging and congenital pathologies (i.e., hypertrophy of the ligaments). The pathogenesis of DCM can be divided into: static, dynamic, and histopathologic factors (Badhiwala et al., 2020). Patients present with a variety of symptoms, such as gait deficits in the lower extremities, as well as loss of manual dexterity, pain, and GI dysfunction (Nouri et al., 2020; Lannon and Kachur, 2021; Davies et al., 2022). The diagnosis currently requires both imaging and clinical assessments, and the treatment option are either non-operative or surgical decompression (Badhiwala et al., 2020).

In the GI tract, gut microbiota plays a key role in shaping and maintaining organ function and eubiosis. Gut microbial ecosystems are composed of bacteria, fungi, viruses, and archaea (Mayer et al., 2022). It communicates with the central nervous system (CNS) through direct and indirect mechanisms that involve interaction with immune cells (Agirman et al., 2021). Gut dysbiosis, defined as the imbalance of the normal gut microbiota characterized by loss of control over mechanisms governing microbial growth in the colon (Lee et al., 2022), has been associated with a number of pathological conditions (e.g., traumatic SCI, stress, Parkinson’s disease, multiple sclerosis, among others) (Kigerl et al., 2016; Sampson et al., 2016; Berer et al., 2017; Marin et al., 2017). Some studies have reported gut dysbiosis following traumatic SCI in both humans and animal models (Gungor et al., 2016; Kigerl et al., 2016; Du et al., 2021). These results vary according to the injury level, sex, and duration of SCI (Gungor et al., 2016; Zhang et al., 2018; Du et al., 2021). It has been shown that in animal models of traumatic SCI, *Bacteroidales* are reduced whereas *Clostridiales* are increased during the first month after injury (Kigerl et al., 2016). At chronic time points, 8 weeks after SCI, *Bifidobacteriales* and *Clostridiales* abundance increased compared to the control group (O'Connor et al., 2018). Furthermore, SCI-induced gut dysbiosis has been associated with an increased abundance of what is thought to be pathogenic microbiota while decreasing non-pathogenic ones, as well as a reduction of beneficial microbial function (Du et al., 2021). Gut dysbiosis has been linked to impaired neurological function by exacerbating the lesion size and neuroinflammation within the spinal cord after SCI (Kigerl et al., 2016). In humans SCI patients (both female and males), the number of butyrate-producing communities (members of the families *Lachnospiraceae* and *Baceteroides*) is reduced compared with healthy patients (Gungor et al., 2016). In male patients with SCI, the abundance of *Bacteroides* has been shown to be higher than that in healthy controls (Zhang et al., 2018).

To date, although gut dysbiosis has been studied in the context of traumatic SCI, it is unknown whether gut dysbiosis occurs after DCM and whether its extent affects the progression of DCM. It has been reported that 16% of DCM patients suffer from GI comorbidities, which can influence both progression and recovery after surgical decompression (i.e., neurologically, psychiatrically, and pain perception) (Nouri et al., 2020). Considering that DCM is more frequent in males than in females (Nouri et al., 2022) and that the gut microbiota can modulate inter-sex differences (i.e., pain sensitivity, diagnosis and prevalence of neurodevelopmental and mental disorders) (Jaggar et al., 2020; Caputi et al., 2022), we sought to deepen our understanding of the role of the gut-brain axis in the pathobiology of DCM.

Here, using a clinically relevant mouse model of DCM (Vidal et al., 2017, 2019), we show for the first time, using 16S rRNA sequencing, that DCM induces changes in the gut microbiome, and that these changes are specific to certain bacterial species. These changes are dynamic over time and more pronounced in males than females. Immune cell composition was also altered in gut-associated lymphoid tissues (GALT) as well as at the systemic level in both sexes. This work contributes to a better understanding of why DCM is more prevalent in males than females, and thus facilitates personalized approaches for diagnosing and managing DCM patients.

## Materials and methods

### Experimental design and DCM induction

**Figure 1:**
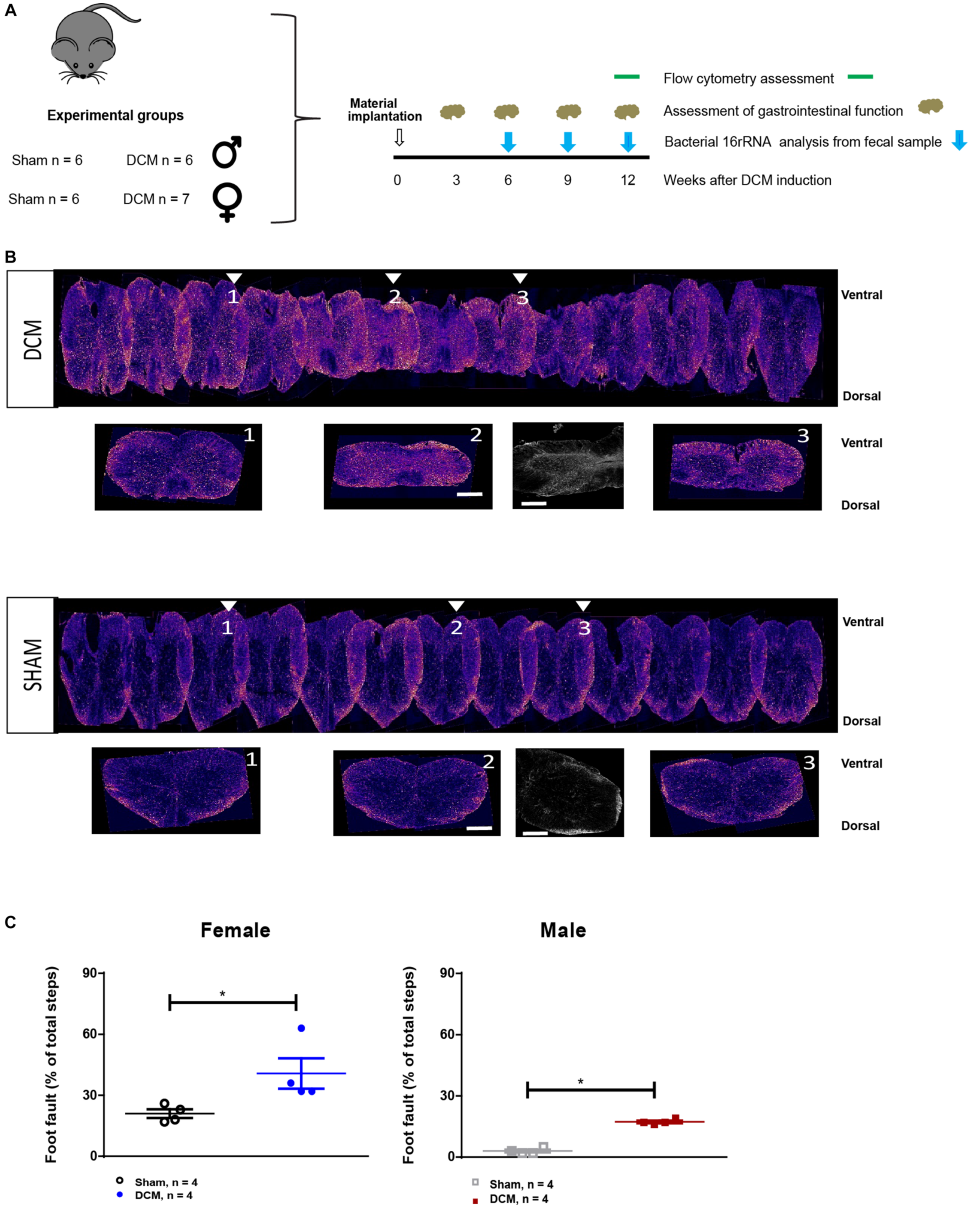
Experimental design. **(A)** Scheme of the experimental design indicating experimental groups and the time points selected for sample collection and the readouts used. **(B)** Representative reconstruction of the DCM (upper panel) and sham (lower panel) spinal cords. The orange indicates astrogliosis (GFAP^+^ staining) in both groups. The arrows show representative coronal sections of three areas, cranial (1), at the epicenter (2) and caudal (3) of the compression area. There is also a higher magnification of GFAP staining of the compression epicenter for the DCM and sham groups (lower panel, in the middle). Scale bar: 400 μm. **(C)** The average percent (%) foot faults in the horizontal ladder walk was significantly increased in the both female and male DCM groups compared with the sham group (*p < 0.05, Mann–Whitney U test).

All surgical and postoperative care procedures were performed in accordance with the Animal Use Committee (AUC) guidelines of the Universidad Católica de la Santísima Concepción, Concepción, Chile. Twelve female and thirteen male C57BL/6 mice (8 weeks old) were purchased from the ISP (Chile) and randomly allocated to either the sham or DCM group. We divided 12 females into sham (*n* = 6) or DCM (*n* = 6) groups, whereas 13 males were divided into sham (*n* = 6) and DCM (*n* = 7) groups. The female and male DCM and sham groups were housed with 2–3 mice per cage. Fecal samples were randomly selected from 11 females and males for bacterial 16S rRNA analysis (Figure 1A) from different cages to avoid all sequenced mice from each group coming from the same cage. Tissue samples from 25 female and male mice were isolated and analyzed using flow cytometry (Figure 1A). The mice were anesthetized with 2% isoflurane, and a midline incision at the C4 level was performed. An aromatic polyether material was implanted underneath the C5–6 laminae to induce chronic and progressive compression of the cervical spinal cord as previously described (Vidal et al., 2017; Ulndreaj et al., 2022). A sham group was included for comparison, in which the aromatic polyether material was inserted under the C5-6 laminae for 30 s, and then removed. Animals within each cage received the same treatment (sham or DCM surgery). All animals received the same feeding diet (PROLAB RMH 3000) and water *ad libitum*, to avoid changes in microbiota composition owing to variations in feeding behavioral (Lee et al., 2022). All animals were housed in a facility with a 12:12 light/dark cycle.

### Fecal sample collection and gastrointestinal function

Fecal samples were collected at 6, 9, and 12 weeks after DCM induction. The mice were removed from their home cage and placed in an empty cage with a cage filter top. Samples were collected over a 30-min period in a sterile tube, and immediately frozen, and stored at −80°C until further processing (Kigerl et al., 2016). Fecal samples were collected in the morning, to avoid confounding differences associated with circadian patterns. Fecal output was measured as previously described. Briefly, mice were individually placed into an empty cage, and fecal pellets were counted over a 15-min period (Sampson et al., 2016). Additionally, the gut length was measured at the end of the experiment.

### Horizontal ladder walk

The ladder walk consisted of two plexiglass plates (69 × 9 cm) connected by 10 rungs, each 1 cm apart as previously described (Ulndreaj et al., 2022). It was used to assess locomotor deficits in females and males. Two trials were video-recorded for each animal, and the foot fault steps out of all steps during each trial were counted. The average (of two trials) percent (%) foot fault steps were plotted for each animal (Ulndreaj et al., 2022).

### Bacterial 16S rRNA sequencing and analysis

Frozen fecal samples were shipped on dry ice to Microbiome Insights Inc. (Vancouver, Canada) for 16S rRNA sequencing. DNA extraction was performed using the MoBio PowerMag Soil DNA Isolation Bead Plate on a KingFisher robot. PCR-based amplicons were generated from the extracted DNA using primers targeting the V4 region of bacterial 16S SSU rRNA (Caporaso et al., 2012). Amplicons were further sequenced using an Illumina MiSeq machine in paired-end mode with 250 base-pair read length (Kozich et al., 2013). Thirty three female and male sequencing datasets were, respectively, processed using the nf-core/ampliseq framework (Straub et al., 2020), implemented on the NextFlow platform (Ewels et al., 2020). After read quality control and filtering using the Phred score, Amplicon Sequence Variants (ASVs) were obtained using the DADA2 pipeline (Callahan et al., 2016). Ribosomal ASVs were predicted using Barrnap.^1^ To distinguish between known and novel taxa and remove unwanted taxa, filtered 16S rRNA sequences were extracted and classified with QIIME2 (Hall and Beiko, 2018) by a combination of a naïve-bayes algorithm and sequence alignment based on BLAST+ (Camacho et al., 2009) and VSEARCH (Rognes et al., 2016), respectively. The classified sequences were taxonomically assigned using the reference database Silva 138.1 prokaryotic SSU database (Pfeiffer et al., 2014). Finally, 744 high-quality ASV sequences were obtained, abundance tables were constructed, and the final results were obtained using MultiQC (Ewels et al., 2016). To obtain Bray-Curtis ordination plots, alpha-diversity estimations via Shannon entropy, and microbial abundance bar plots, respectively, DADA2 output tables were imported into R language and processed using the phyloseq R package (McMurdie and Holmes, 2013).

### Phylogenetic tree construction

The 744 high-quality ASV sequences were aligned using the MAFFT multiple sequence alignment program v7.490 using the –reorder flag (Katoh et al., 2002, 2009). A phylogenetic tree was then constructed using FastTree software v2.1 using the MAFFT output alignments FASTA format as input along with the -nt flag. Edition and plot of the phylogenetic tree were generated using the Interactive Tree of Life server (iTOL), collapsing all clades whose average branch length distance was below 0.0002 (Letunic and Bork, 2007).

### Differentially abundant ASVs

Differentially abundant ASVs between sham and DCM conditions were obtained by reading the DADA2_table.tsv into the R software and employing the R edgeRun package to interrogate ASV differentially abundant between sham and DCM conditions across sexes (Dimont et al., 2015). We considered significantly differentially abundant ASVs with a fold change over 0.5 and a False Discovery Rate (FDR) less than 0.05.

### ASVs to genomes assignments

We aimed to match every differentially expressed ASV from the 16S rRNA sequencing experiment to a candidate microbial genome using the iMGMC mouse-gut dereplicated metagenome-associated genomes collection (iMGMC-hqMAGs-dereplicated_genomes.tar.gz) containing 132,958 contigs distributed across 830 high quality microbial genomes (N50 = 40,316, 2,396 Mb) (Lesker et al., 2020). A BLAST database was constructed containing these genomes and employing BLASTn with a cutoff of 95% to match an ASV to a given genome (Camacho et al., 2009). The BLASTn results were parsed using a script that relies on the biopython library (Cock et al., 2009) and SeqKit tool (Shen et al., 2016)^2^ and microbial MAGs matching ASVs were parsed and extracted from the complete iMGMC fasta collection. Using this procedure, we matched 17 MAGs with male ASVs and seven MAGs with female ASVs.

### Anvi’o processing and taxonomic identification of metagenomes-assembled genomes

The identified metagenomes-assembled genomes (MAGs) from male and females ASVs were merged as single metagenomes, respectively (hereafter, male and female metagenome), and each metagenome was processed using the Anvi’o platform for metagenomics (hope, v7.1) (Eren et al., 2015). Per the metagenome, a separated contig database was constructed, and then we profiled k-mer frequencies, functional annotation of open reading frames (ORFs), and GC content of every contig, respectively. We initially binned contigs exactly as in the MAGs collection and further refined these bins by manual human-guided binning to obtain >90% completion and < 10% redundancy values per bin. Binning refinement is not necessary for the female metagenome, given that both the completion and redundancy values for the female metagenome had already met the defined criteria during the MAGs creation process in Anvi’o, a process that involved contig separation and clustering based on tetranucleotide frequency and phylogenetic differentiation, the binning refinement was not necessary. In contrast, the male metagenome contained a higher number of MAGs than the female, suggesting greater microbial diversity and complexity in the male sample. As a result, the male metagenome required manual bin refinement. After refining the bins in the male metagenome, we identified the taxonomy of each bin according to the closest match with genomes from the GTDB database, consisting of 317,542 genomes (database release 07-RS207, 8th April 2022) (Chaumeil et al., 2022). Metaproteomes were resolved using prodigal v2.6.3 (Hyatt et al., 2010). We assessed MAG redundancy and completion using the CheckM tool (Parks et al., 2015). In each MAG, genes and proteins were annotated from each metagenome using NCBI’s Clusters of Orthologous Groups (COGs) database (Tatusov et al., 2000).

### Pangenome reconstruction of metagenomes

In both the female and male metagenomes, we employed the anvi’o function “anvi-get-sequences-for-gene-calls” with the flag --export-gff3 to export a single gff file belonging to each metagenome. Each gff file was parsed by the MAG using a modified bash script available at: https://groups.google.com/g/anvio/c/DWO8fDQ_g7M. In this manner, in both female and male metagenomes, we obtained gene calls per MAG in gff format, which were placed in a given folder and served as input to construct a pangenome matrix via Roary, using the following command: roary -e --mafft -p 30 (Page et al., 2015). As described in the Roary README, each pangenome matrix was plotted using the roary_plots.py script using as inputs the MAG phylogenetic tree (accessory_binary_genes.fa.newick) and gene_presence_absence.csv tabular file, both outputs of Roary.

### Metabolic genes and network reconstruction

To identify metabolic genes along metagenomes, we re-annotated all proteins from male and female metagenome databases using the KEGG ortholog assignment KOfam method (Aramaki et al., 2020), including the use of the KEGG BRITE Database (Kanehisa et al., 2017). Enriched metabolic modules were obtained by determining the presence of a given metabolic module in MAGs associated with sham or DCM and quantifying the distribution of this module across all MAGs belonging to a given metagenome. A module was considered to be enriched in sham or DCM-associated MAGs if the q-value of the Rao score test was below 0.05, as described here (Shaiber et al., 2020). The latter function is fully implemented in the anvi’o function “anvi-compute-metabolic-enrichment.” Statistically significant modules were plotted as Sankey plots using the R package network3D.^3^ The entire metabolic reconstruction was benchmarked using the gapseq program (Zimmermann et al., 2021). Contigs from all MAGs were annotated using a curated reaction database composed of the MetaCyc (Caspi et al., 2016), KEGG (Kanehisa et al., 2019) and ModelSEED (Henry et al., 2010) databases. Prediction of metabolic pathways and gap-filling per MAG was performed by running the gapseq_find.sh script with the following flags: -v 0 -b 200 -p all -t auto. To model the influx and efflux of metabolites, we chose as gapfill-medium a formulation made of glucose and a minimal amount of acetate, with no amino acids, into a BacArena modeling framework, as described here: https://bacarena.github.io/ (Bauer et al., 2017). The chosen parameters for the simulations were as following: arena size of 50 × 50 grid cells, random population of two grid cells as starting culture, removal of acetate from the initial substrate list allow the community to grow with bacterial acetate from cross-feeding, and performing simulations for 14-time steps, respectively. Parsing of all reconstructed pathways, including text-processing, was executed through the Jupyter Notebook using libraries such as NumPy (Harris et al., 2020) and pandas.^4^

### Flow cytometry

Blood samples and cells from the spleen and colonic lamina propria, were collected and analyzed by flow cytometry 12 weeks after DCM induction, as previously described (Ulndreaj et al., 2016; Vidal et al., 2017; Ugalde et al., 2021). Red blood cells from the spleen and blood samples were lysed in red blood cell lysis buffer ACK (Thermo Fisher) for 5 min and viable cells were stained with a viability dye (Fixable viability dye Red 780, TonboBio) for 20 min. The following antibodies were used to distinguish granulocytes, monocytes, dendritic cells, and T lymphocytes: Ly6C (clone HK1.4) conjugated to PerCP/Cyanine5.5, Ly6G (clone 1A8) conjugated to APC, CD11b (clone M1/70) conjugated to FITC, CD3 (clone 17A2) conjugated to PerCP/Cyanine5.5, CD8 (clone 53–5.8) conjugated to FITC, CD4 (clone GK1.5) conjugated to APC, CD11c (clone N418) conjugated to FITC, and CD25 (clone 3C7) conjugated to PE (BioLegend, San Diego, United States). Matching isotype controls or fluorescence minus one (FMO) was used to gate the positive cells. Data were acquired using a BD FACS Canto II (BD Biosciences, San Jose, CA, United States) and analyzed using FlowJo X 10 (BD Biosciences, Ashland, United States). Samples that did not meet the minimum number of events (100) were excluded from analysis.

### Histological analysis

The animals were transcardially perfused with phosphate buffered saline (PBS). The spinal cords were dissected out (0.3 cm rostral and 0.3 cm caudal from the compression epicenter), and post-fixed with 4% paraformaldehyde (PFA) in PBS, and cryoprotected in 30% sucrose/PBS for 48 h. Coronal sections (20 μm thick) were prepared and blocked (5% normal serum and 0.3% triton X-100 in PBS) for 1 h at room temperature (RT). Incubation with the primary antibody GFAP (1,1,000, C9205, Cy3-conjugated, Sigma) and 4′,6-diamidino-2-phenylindole (DAPI, 1:200, Sigma), was performed at RT for 1 h. Sections were systematically sampled every 580 μm over 3,420 μm (13 sections per animal). Reconstruction was performed using ImageJ software bundled with 64-bit Java 8 (Schneider et al., 2012) and the related Fiji distribution (Schindelin et al., 2012) using built-in plugins, including the MosaicJ external plugin.

### Statistical analysis

All statistical analyses were performed using the GraphPad Prism 5.01 software (GraphPad Software, Inc.). Data sets were analyzed for normal distribution using the D’Agostino-Pearson normality test. GI function was analyzed using two-way analysis of variance (ANOVA) for repeated measurements with Tukey’s multiple comparison test. Flow cytometry analyses and ladder walk test results were analyzed using the nonparametric Mann–Whitney U test. Data are presented as mean ± standard error of the mean (SEM). Differences were considered statistically significant at *p* ≤ 0.05.

## Results

### Changes in white blood cells are more pronounced in females than males

To characterize the systemic changes in the blood during DCM, we divided the animals into four groups that received either sham or DCM surgery (Figure 1A). We used a previously characterized DCM mouse model that resembles the human pathology (Vidal et al., 2017; Laliberte et al., 2021; Ulndreaj et al., 2022). At 12 weeks post DCM-induction there is a 47% compression ratio in the mouse DCM model (Vidal et al., 2017), loss of motor neurons (Satkunendrarajah et al., 2018; Ulndreaj et al., 2022), and development of motor impairment (Vidal et al., 2017; Laliberte et al., 2021; Ulndreaj et al., 2022). Representative images of reconstructed spinal cords of sham and DCM groups after DCM confirmed spinal compression versus sham (Figure 1B, upper and lower panels), as well as increased astrogliosis (Figure 1B, upper and lower panels, small inserts), and locomotor deterioration (Figure 1C) resembling previously published findings from the DCM mouse model (Vidal et al., 2017; Desimone et al., 2021).

**Figure 2:**
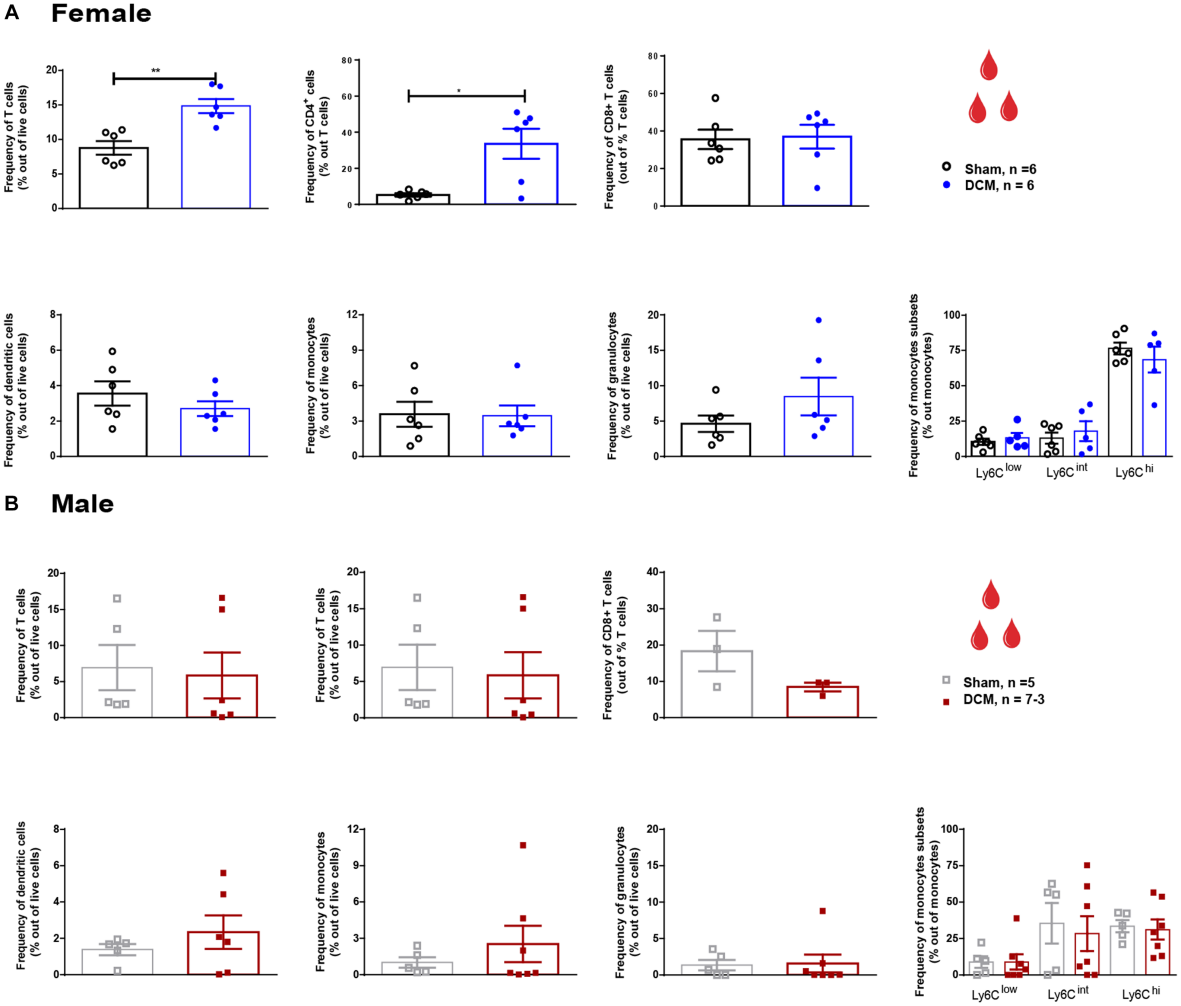
Systemic cellular changes following DCM in females and males. Flow cytometric quantification of the frequency of circulating T cells (CD3^+^), CD4^+^ T cells, CD8^+^ T cells, dendritic cells (Cd11c^+^), monocytes (Ly6C^+^CD11b^+^Ly6G^−^), neutrophils (Ly6G^+^CD11b^+^Ly6C^−^), and monocyte subsets Ly6C ^low^, Ly6C ^int^, and Ly6C ^hi^ at 12 weeks after DCM induction in females **(A)** and males **(B)**. All values are reported as frequency out of live cells. Asterisks indicated significant differences between sham and DCM groups. Females: Sham = 6; DCM = 6; Males: Sham = 6; DCM = 7–3. Data are presented as mean ± SEM. *p ≤ 0.05; **p ≤ 0.01.

At the systemic level, T cells in DCM females presented a 1.7-fold increase compared to the sham group (Figure 2A; **p = 0.0022) and a 6-fold increase in the frequency of CD4^+^ T cells (Figure 2A; *p = 0.0411). In males, no significant changes were detected at the systemic level in the T cells (Figure 2B). Non-significant changes between the DCM and sham groups were observed in the CD8^+^ T cell subset, dendritic cells, granulocytes, monocytes, and monocyte subsets in both females and males (Figures 2A,B, respectively).

### DCM leads to changes in the immune cell composition of the gut-associated lymphoid tissues

**Figure 3:**
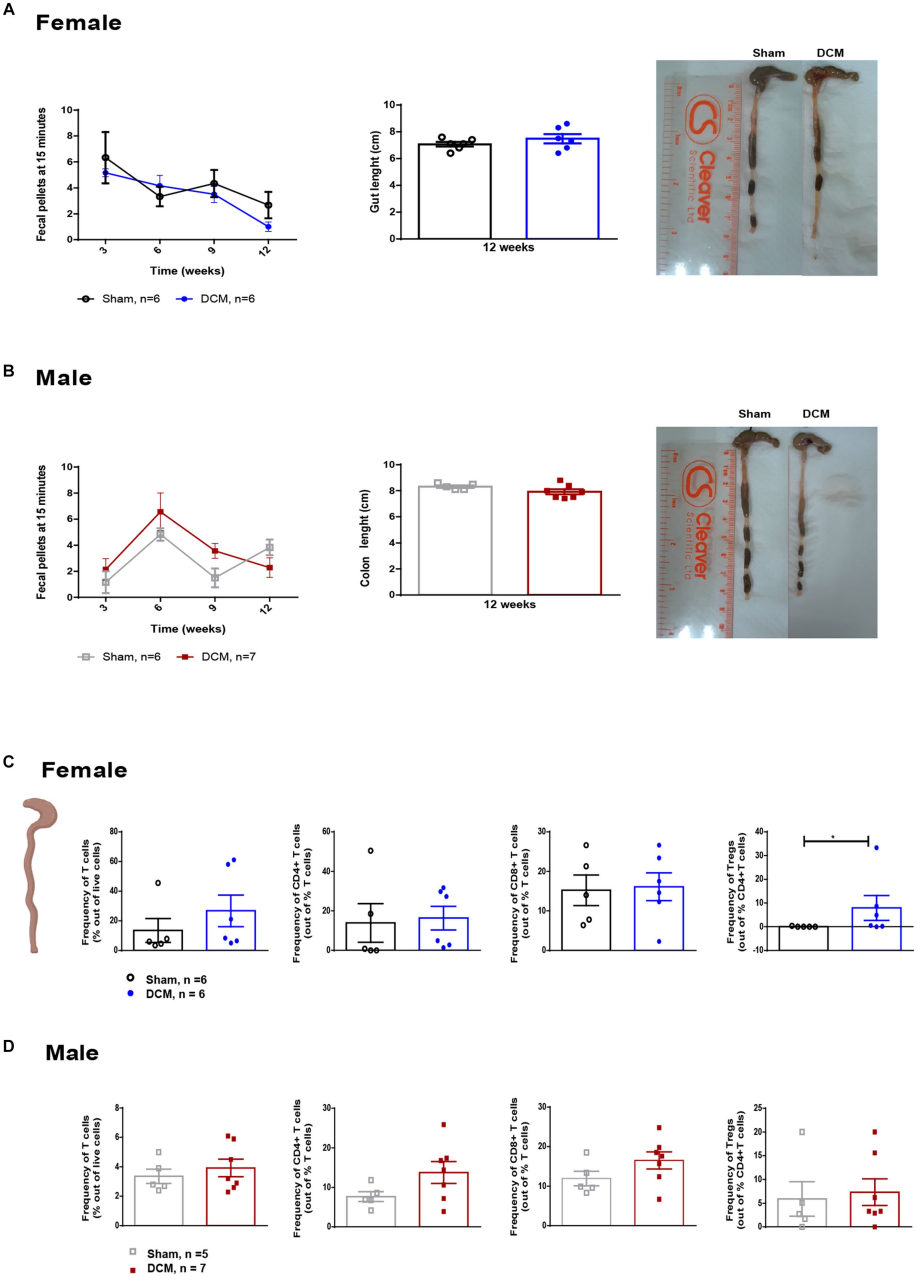
Gut function and immune composition following DCM. Time course of fecal output in a novel environment over 15 min period in females (**A**, left panel) and males (**B**, left panel). Quantification of colon length at 12 weeks after DCM induction as well as representative images for sham and DCM females (**A**, right panel) and males (**B**, right panel). Females: Sham = 6; DCM = 6; Males: Sham = 6; DCM = 7. Flow cytometric quantification of the frequency T cells (CD3^+^), CD3^+^CD4^+^CD8^−^ T cells, CD3^+^CD4^−^CD8^+^ T cells, and Tregs (CD3^+^CD4^+^CD25^+^) at 12 weeks after DCM induction in females **(C)** and males **(D)**. Females: Sham = 6; DCM = 6; Males: Sham = 5; DCM = 7. Data are presented as mean ± SEM. **p ≤ 0.01.

**Figure 4:**
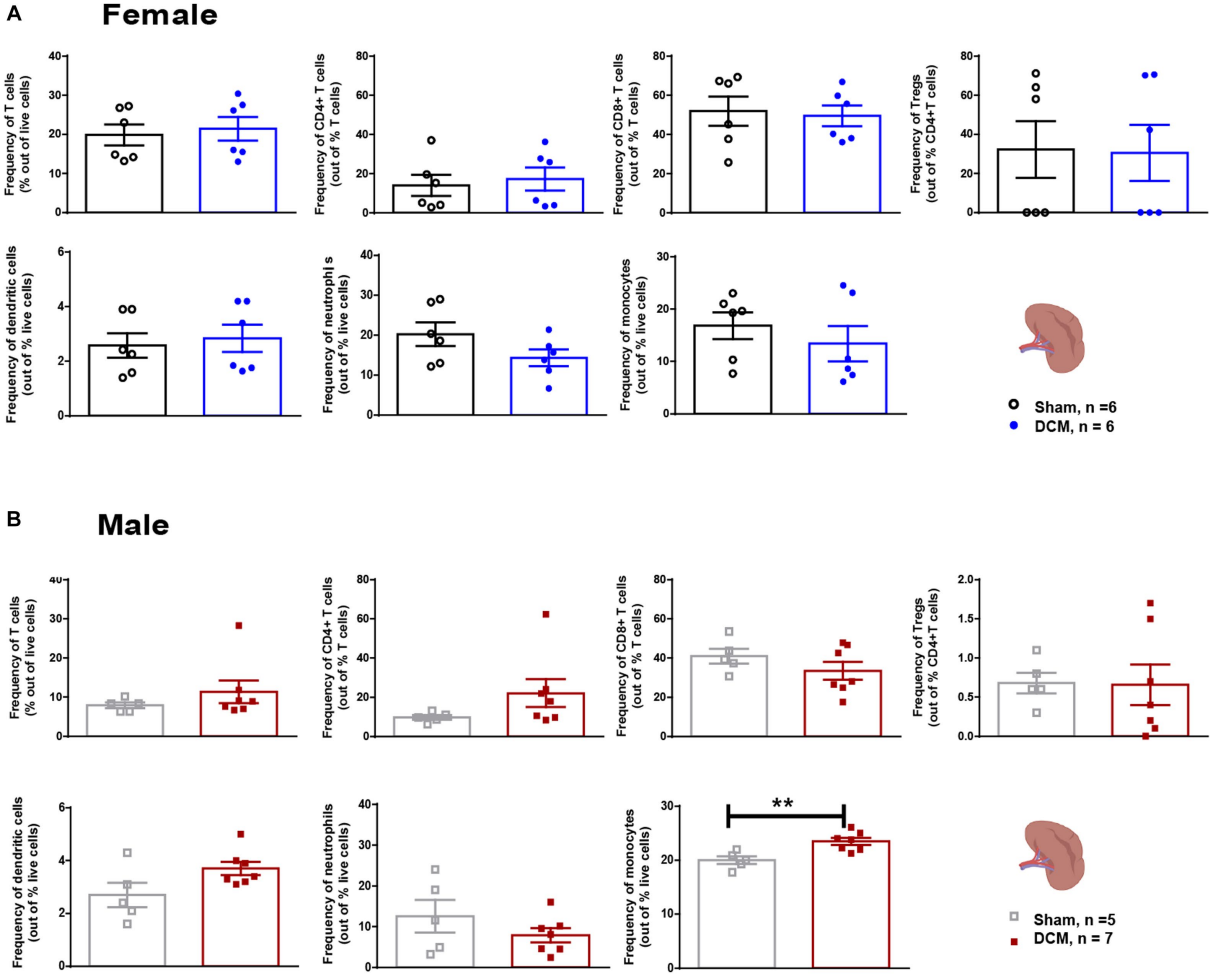
Spleen cellular composition after DCM. Flow cytometric quantification of the frequency of T cells (CD3^+^), CD4^+^ T cells, CD8^+^ T cells, Tregs (CD3^+^CD4^+^CD25^+^), dendritic cells (Cd11c^+^), granulocytes (Ly6G^+^CD11b^+^Ly6C^−^), and monocytes (Ly6C^+^CD11b^+^Ly6G^−^), at 12 weeks after DCM induction in females **(A)** and males **(B)**. Females: Sham = 6; DCM = 6; Males: Sham = 5; DCM = 7. Data are presented as mean ± SEM. *p ≤ 0.05.

To assess gastrointestinal function and constipation in our DCM mouse model, we analyzed the total output of fecal pellets and the total colon length in the sham and DCM groups. We did not observe significant differences neither in the time course of fecal output over a 15-min time period or the colon length between the sham and DCM groups in females (Figure 3A) or males (Figure 3B). The gut wall is normally composed of a layer of epithelial cells (enterocytes), secretory cells, and the GALT. The latter mainly uses lymphocytes to carry on immune responses (Mowat and Agace, 2014) and communicates with the CNS by interacting with nerve fibers, secreting metabolites, and immune cells (Mowat and Agace, 2014). We previously reported that systemic T cell levels vary during DCM progression and following surgical decompression (Vidal et al., 2018; Ulndreaj et al., 2022). Thus, to evaluate the effect of DCM on T lymphocytes, we measured the frequency of T lymphocytes and three different subsets, CD4^+^ T cells, CD8^+^ T cells and regulatory T cells (Tregs) in the colonic lamina propria and spleen. There were no significant differences in the frequency of T lymphocytes, CD4^+^ T cells, and CD8^+^ T cells between the sham and DCM groups in both female and males. However, DCM females showed a significant increase (*p = 0.045) in the frequency of Tregs compared to sham females (Figure 3C). Non-significant differences were observed in males (Figure 3D). Similarly, in the spleen, T cells, CD4^+^ T cells, CD8^+^ T cells, and Tregs had similar frequencies between the sham and DCM groups in both females and males (Figures 4A,B). In the myeloid family, dendritic cells, neutrophils, and monocytes reached comparable levels in females (Figure 4A), whereas in males the frequency of monocytes there was a 1.2-fold increase (**p = 0.0076) in the DCM group compared with sham males (Figure 4B).

### DCM-induced dysbiosis is sexually dimorphic

To investigate the effects of DCM on the mouse gut microbiota, we hypothesized that DCM causes gut dysbiosis in a mouse model. To address the latter, we designed an experimental approach in which males and females were randomized into two groups, either sham or DCM (Figure 1A). Fecal samples were longitudinally collected from the same animal at three selected time points: 6-, 9-, and 12-weeks post-DCM induction, herein referred to as early, mid, and late, respectively. The selected time points: early, mid, and late, were chosen based on previous work with the DCM mouse model that showed the presence of symptomatology (e.g., manual dexterity impairment, pain, and locomotor/gait deficits) (Vidal et al., 2017; Laliberte et al., 2021; Ulndreaj et al., 2022). To further characterize the gut microbial community in both females and males during DCM progression, we employed a 16S rRNA sequencing approach, in which DNA was extracted from fecal samples and the bacterial 16S SSU rRNA V4 region was amplified by PCR and further sequenced using Illumina technology, as described in methods section. As a control, we included a sham group for each week of dysbiosis. We sequenced five control samples and six treatment samples per week and per sex, respectively obtaining 66 samples in total (see Supplementary Table S1) which were input into the NextFlow ampliseq nf-core pipeline. First, DADA2 (Callahan et al., 2016) constructed a probabilistic model, based on the inferred sequencing error rates of reads that were used to denoise and collapse these reads as contigs, denominated Amplicon Sequence Variants (ASVs, see Supplementary Figure S1).

**Figure 5:**
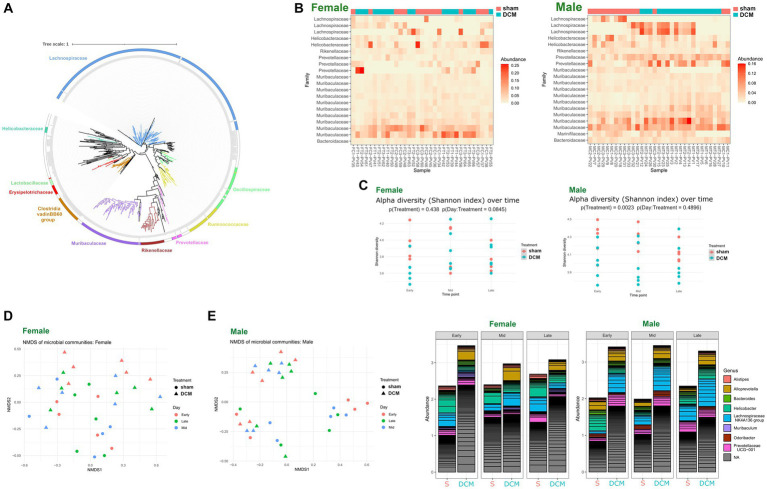
DCM-induced dysbiosis is sexually dimorphic. **(A)** Phylogenetic tree constructed with 744 high-quality ASV sequences derived from all female and male 16S sequencing samples. Sequences were aligned using the MAFFT multiple sequence alignment software and a maximum likelihood phylogenetic tree was constructed using Figtree software. Plotting and editing of the phylogenetic tree was performed using the Interactive Tree of Life (iTOL) server. The scale of the tree was shown above. **(B)** ecologically organized heatmap plot of all females (upper) and male (lower) samples abundance across phylogeny (at the family level), resolved by phyloseq. Samples corresponding to Sham or DCM were colored magenta and turquoise, respectively. **(C)** Alpha diversity (Shannon index) over time in the sham and DCM groups across male and female sequencing samples. The plot displays the Shannon diversity index at early, mid, and late time points for both sham (magenta) and DCM (turquoise) groups. Points represent individual samples. The results of a two-way ANOVA test are displayed under the title, indicating no significant (*p* > 0.05) or significant (*p* < 0.05) interaction effect between time and DCM. **(D)** Non-metric multidimensional scaling (NMDS) plot of microbial community composition in the sham and DCM groups across males and females sequencing samples. The plot displays the NMDS1 and NMDS2 axes, which represent the dissimilarity in microbial community composition between samples. Each point represents an individual sample, with sham samples shown in red and DCM samples shown in blue. **(E)** Bar graphs of microbial abundance of the top 20 main genera detected in female (left) and male (right) samples, respectively. Samples were grouped into two levels: sham or DCM and whether they belong to early, mid, and late time points (6, 9, and 12 weeks after DCM induction). ASV abundance was colored by genus, and ASVs that did not have taxonomy assignation at the genus level, were classified as NA.

Phylogenetic assignament of non-redundant ASVs by DADA2, revealed the presence of different genera, particularly the bacterial members of *Lachonospiraceae*, *Muribaculaceae*, *Oscillospiraceae*, *Ruminococcaceae,* and *Clostridia*, among other families, respectively (Figure 5A and Supplementary Table S2). We plotted the abundance of each family across female and male samples, respectively using phyloseq ecologically-oriented ordination methods, based on the NeatMap approach (Rajaram and Oono, 2010). Notably, females did not show a significant difference in alpha diversity between sham and DCM groups, but males did: a clear separation between sham and DCM samples was not evident across female samples when abundances were plotted and ordered across families (Figure 5B, upper heatmap). In contrast, a clear ordination between sham and DCM samples was evident across male samples, when abundance was plotted across all detected families (Figure 5B lower heatmap, respectively). In females, alpha diversity was not significantly different between the sham and DCM groups (*p* = 0.438), with a tendency of time effect (Figure 5C, upper panel, *p* = 0.0845). However, a trend is observed at the early time point, where the Shannon diversity is higher in the sham group compared to DCM (p (Day:Treatment = 0.0845)). In males sham and DCM groups presented a clear separation (Figure 5C, lower panel, *p* = 0.0023) without a time effect (*p* = 0.4896). Although the time effect was not significant, there is a clear trend where Shannon diversity decreases over time in the sham group, while it increases in DCM. Consistent with the latter, samples ordered according to the Bray–Curtis dissimilarity in a two-dimension Non-metric Multi-dimensional Scaling (NMDS) plot, revealed spatially mixed sham and DCM samples in females, respectively (Figure 5D, left). In contrast, the male sham and DCM samples were spatially ordered with clear separation in the same plots (Figure 5D, right). Figure 5E shows bar plots of the relative abundance of microbial populations belonging to the top 10 genera across sex and disease progression (early, mid, and late DCM, respectively). A clear increase in the abundance of bacterial members belonging to *Alloprevotella*, *Lachonospiraceae,* and *Helicobacter* genera was observed across DCM states in both sexes, but the bacterial population dynamics were different between male and female DCM. In females, early after DCM induction, bacterial members belonging to genera such as *Alloprevotella*, *Helicobacter,* and *Muribaculum* increased in abundance, whereas *Alistipes* genus diminished in DCM compared to the sham group, except in the mid DCM condition (Figure 5E, left panel). Importantly, the abundance of *Lachnospiraceae NK4A136* genus increased during DCM progression at mid and late time points compared to that in the control group (Figure 5E left panel). In contrast, changes in abundance in males were more pronounced than in females: the abundance of bacterial members belonging to *Alloprevotella*, *Bacteroides, Helicobacter, Odoribacter*, *Lachnospiraceae NK4A136,* and *Prevotellaceae UCG-001* consistently increased in the DCM group compared to the sham group at all time points (Figure 5E, right panel). Overall, these findings confirm that dysbiosis induced by DCM is sex specific. Although there is a general effect of increased abundance of *Alloprevotella* and *Lachonospiraceae* genus in both sexes, more pronounced bacterial abundance changes were observed in male gut microbiomes. In conclusion, our initial analysis in both sexes showed significant changes in the composition of the murine gut microbiota between sham vs. DCM, indicating that DCM induced sexually dimorphic dysbiosis in mice.

### Differential abundance of bacteria following DCM in male and female gut microbiomes

**Figure 6:**
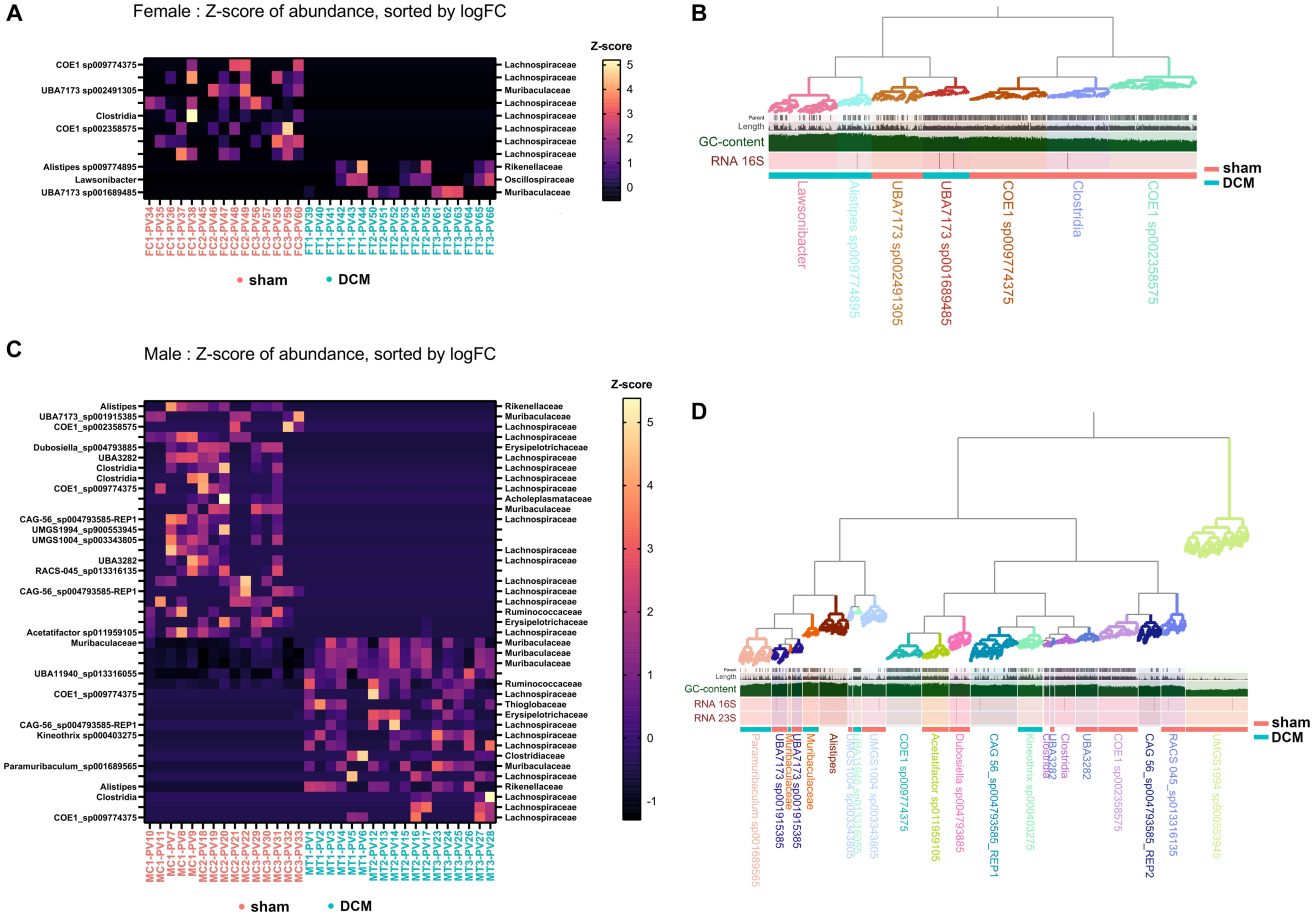
Differential abundance of bacteria following DCM in male and female gut microbiomes. **(A)** Abundance plot of 11 ASV significantly abundant in sham versus DCM comparison, across female samples. The Z-score of the abundances was plotted on the heatmap. Samples were ordered on the x-axis whether they belong to the sham (magenta) or DCM (turquoise) condition and on the y-axis by the -log10 fold change in the sham vs. DCM comparison, obtained with edgeRun. In the heatmaps, from top to bottom, correspond to less to greater abundance in the DCM condition. On the right of the plot, we show the matching metagenome-associated genome (MAG) with >95% identity to a given ASV, identified by BLASTn and using the iMGMC dereplicated genome collection as database. **(B)** Anvi’o female MAGs metagenome reconstruction based on iMGMC MAGs matching female ASVs from **(A)** with >95% identity. The metagenome-assembled genomes are highlighted with different colors and clustered according to GTDB taxonomy classification systems (>50% completion, <10% redundancy). Each contig was hierarchically plotted according with its tetranucleotide frequency and GC content altogether. MAGs present in sham or DCM condition were identified with a magenta or turquoise bar, respectively. **(C)** Same as **(A)** for the 41 differentially abundant ASVs in male samples when compared sham vs. DCM condition. **(D)** same as **(B)** for iMGMC MAGs matching the male ASVs from **(B)** with >95% identity.

We aimed to closely examine the bacterial changes caused by DCM in both male and female gut microbiomes. Our goal was to link each ASV identified during the 16 s rRNA sequencing with a microbial genome from the mouse gut. To achieve this, we used the mouse-gut dereplicated metagenome-associated genomes collection as our reference (iMGMC-hqMAGs-dereplicated_genomes.tar.gz) as the genomic database, a genome collection in FASTA format consisting of 132,958 contigs distributed across 830 high quality microbial genomes (N50 = 40,316, 2,396 Mb) (Lesker et al., 2020). Our primary data source was the DADA2_table.tsv file, which lists all ASV sequences from both sham and DCM conditions in both sexes. For this purpose, we employed a mouse-gut dereplicated metagenome-associated genomes collection. We, then we used the *edgeRun* package implemented in R to identify statistically significant ASVs that were differentially abundant when compared to sham versus DCM in both male and female samples. Specifically, we found 11 and 41 such ASVs in female and male samples, respectively (see Supplementary Table S3). BLAST results of these ASVs against the iMGMC contig collection revealed seven and 17 unique genomes with ≥95% 16S identity across female and male samples, respectively (Figures 6A,C, respectively, Supplementary Table S4). Using the anvi’o platform for metagenomics along with the GTDB database as a taxonomic reference (October 2022) we calculated N50, percentage of completion, and redundancy, among other parameters across male and female genomes, respectively (Supplementary Table S4). The percentage of completion of male genomes ranged from ~93% with a median N50 of ~38,000 while the percentage of completion of female genomes ranged from ~94% with a median N50 of ~107,000. Importantly, several female genomes belonging to *Lachonospiraceae* family were abundant in sham but not in DCM and bacteria belonging to *Rikenellaceae*, *Oscillospiraceae*, and *Muribaculaceae* families were exclusively present in female DCM samples, respectively (Figure 6A). GC content, tetranucleotide frequency and taxonomy clustering also supported this observation revealing three *Lachonospiraceae* genomes present in sham but not in DCM that clustered together and contained lower GC content than bacteria present only in DCM condition (Figure 6B). Of note, one of the two bacteria belonging to the *Muribaculaceae* family was present in DCM but not in sham (UBA7173 sp002491305), while the bacteria UBA7173 sp001689485 was present in sham but not in DCM (Figure 6B).

The latter observations can be applied to male genomes, with a clear presence of bacterial members belonging to *Lachonospiraceae* family present in sham, but not in DCM. Conversely, several members of *Muribaculaceae* family were exclusively present in the DCM group, but not in the sham group (Figure 6C). In males, the phylogenetic separation between bacteria presents in sham but not DCM (and vice versa) was not obvious, but bacterial members of *Muribaculaceae* family including the *Paramuribaculum* genus, abundant in DCM, tended to have a higher GC content than bacteria from *Lachonospiraceae* family that is present in sham but not in DCM (Figure 6D). As observed in females, a male bacterium belonging to *Muribaculaceae* family, genus UBA7173 (UBA7173 sp001915385), was present in the sham but not in the DCM condition, whereas two bacteria belonging to the same *Muribaculaceae* family (Paramuribaculum sp001689565 and another bacterium without assigned genus) were present in DCM, but not in sham condition (Figure 6D).

In conclusion, there’s a noticeable shift in GC-rich bacteria from the *Muribaculaceae* family, present in DCM but not in the sham condition across both sexes, whereas several bacterial members of *Lachonospiraceae* along with other families tended to be absent when DCM-induced dysbiosis prevailed. Of note, in males, bacteria belonging to *Muribaculaceae* family were absent in the initial phyloseq analysis, because the ASVs associated with these bacteria did not have an assigned genus (Supplementary Table S4).

### Functional characterization of male and female bacteria following DCM-induced dysbiosis

To discover conserved gene clusters of genes associated with bacteria present in DCM-induced dysbiosis, we obtained all gene calls from male and female metagenome in gff format in and we parsed gene calls from each MAG. In this manner, we obtained all genes belonging to each MAG in gff format, respectively (see “Pangenome reconstruction of metagenomes” in Materials and Methods section). Then, in both male and female metagenomes, we calculated each pangenome using the Roary pipeline (Page et al., 2015) using gene calls from all MAGs belonging to a given metagenome. From Figure 6A, we considered four and three MAGs in females exclusively present in sham and DCM condition, respectively while from Figure 6B, we considered eight and four MAGs in males exclusively present in sham and DCM condition, respectively (see Supplementary Table S5, sections “female_venn” and “male_venn,” respectively).

**Figure 7:**
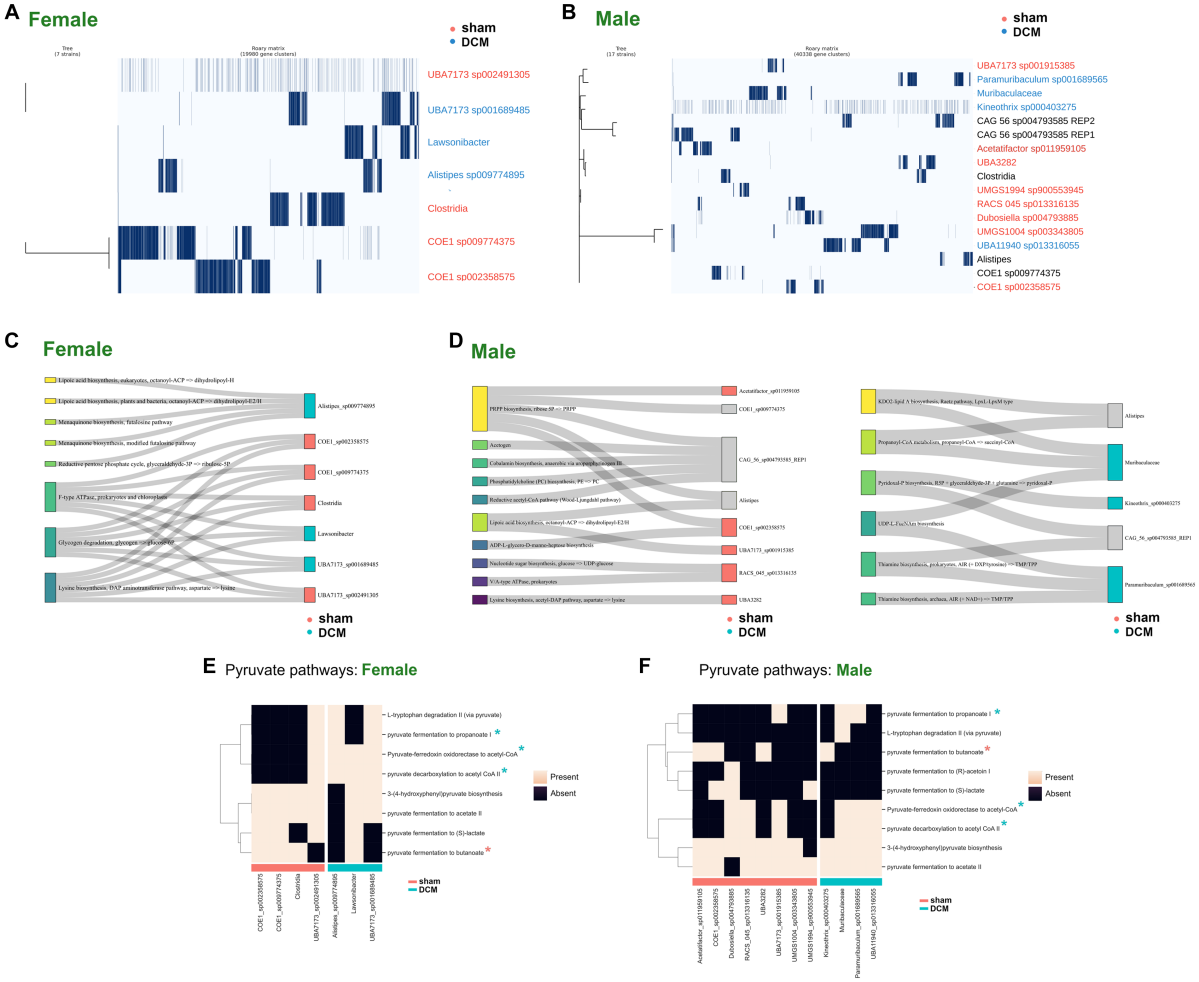
Functional characterization of male and female bacteria following DCM-induced dysbiosis. **(A)** Pangenome plot of cloud and shell gene modules identified by Roary across all female MAGs that correspond with differentially abundant female ASVs with >95% identity in the comparison sham vs. DCM. The samples were ordered according to the phylogenetic tree and MAGs belonging to sham condition were colored in magenta while MAGs belonging to the DCM condition were colored in blue. **(B)** Same as **(A)** for male samples. Samples not uniquely belonging to sham or DCM condition were labeled in black. **(C)** Sankey plots depicting statistically significant pathways found across female MAGs. MAGs belonging to sham condition were colored at right in magenta and MAGs belonging to DCM condition were colored at right in turquoise. **(D)** Same as **(C)** by male MAGs present in only in sham condition (left) or DCM condition (right). **(E)** Heatmap plot depicting presence (light pink color) or absence (black color) of pyruvate-related pathways in sham or DCM-associated female MAGs. MAGs associated with sham were plotted together and denoted with a magenta bar, while MAGs associated with DCM condition were also plotted together and denoted with a turquoise bar. In the y-axis, pathways were hierarchically clustered according to presence or absence value. **(F)** Same as **(E)** for male MAGs.

In the context of pangenomics, the core genome refers to genes that are present in all strains of a set of analyzed genomes, highlighting the essential functions. On the other hand, the soft-core genome, includes genes found in a high percentage, typically over 95%, of the strains (these might be essential for most but not all strains). Lastly, the cloud genome encompasses genes present in a small subset of the analyzed strains, usually fewer than 15%. These genes shed light on the unique adaptations and potential functionalities of certain strains. In our analysis, almost all gene clusters fell under the umbrella of the cloud genome and none of them were classified as soft-core or core genomes in both male and female pangenomes, despite the close phylogenetic relationship of some MAGs belonging to the families *Lachonospiraceae*. and *Muribaculaceae*, present in both pangenomes (see Figures 7A,B, for female and male pangenome matrix, respectively and Supplementary Table S5, sections “common_set_males” and “common_set_females,” respectively). Nevertheless, male MAGs belonging to *Muribaculaceae* family and *Paramuribaculum* genus associated with DCM-induced dysbiosis, presented several conserved genes involved in pyruvate pathways (see Supplementary Table S5, section “common_set_males”).

Since we did not find common gene clusters within MAGs associated with DCM-induced dysbiosis in males and females, we asked whether metabolic pathways were common in MAGs present in DCM. Using the anvi’o genomic annotation per genome as starting point, we re-classified each gene using the KEGG ortholog assignment KOfam method (Aramaki et al., 2020), relying on the KEGG BRITE Database (Kanehisa et al., 2017). Then, we detected enriched metabolic modules in MAGs associated with sham or DCM using the anvi’o function “anvi-compute-metabolic-enrichment” as described in Material and Methods, section “Metabolic genes and network reconstruction.” In females, several statistically significant pathways within MAGs associated with DCM were found, including those related to lipid metabolism and the reductive pentose phosphate cycle (Alistipes sp009774895), F-type ATPase, glycogen degradation, and lysine biosynthesis (Lawsonibacter, UBA7173 sp001689485). Notably, the latter three pathways were also present in MAGs associated with sham (see Sankey plot of Figure 7C, and Supplementary Table S6).

In males, more statistically significant pathways were identified. Pathways related to Phosphoribosyl pyrophosphate (PRPP) biosynthesis, ADP-L-glycero-D-manno-heptose biosynthesis, and Nucleotide sugar biosynthesis, among others, were associated with MAGs preferentially present in sham rather than DCM (see Sankey plot of Figure 7D, *left* and Supplementary Table S7). Regarding pathways present in MAGs associated with DCM-induced dysbiosis, pathways related to lipid A biosynthesis and UDP-L-FucNAm (UDP-N-acetyl-beta-L-fucosamine) biosynthesis were enriched in a MAG belonging to *Muribaculaceae* family, both pathways leading to the biosynthesis of Lipopolysaccharide (LPS) (Mulrooney et al., 2005; Emiola et al., 2014). In addition, the production of Propanoyl-CoA metabolism is exclusively found in this MAG. A pathway involving Thiamine biosynthesis involved in bacterial butyrate production (Soto-Martin et al., 2020; Park et al., 2022) was enriched in *Paramuribaculum sp001689565* and a pathway related to the production of Pyridoxal phosphate biosynthesis enriched in Kineothrix sp000403275 is associated with pathogenesis (Dick et al., 2010; El Qaidi et al., 2013) (see Sankey plot of Figure 7D, right and Supplementary Table S7). This evidence suggests that in both sexes, several pathways are involved in DCM-induced dysbiosis, but other common molecular mechanisms may be present.

To address this, we employed the gapseq program to combine the identified metabolic pathways along metabolic network reconstruction using gap-filling reactions, as described in Materials and Methods (Zimmermann et al., 2021). Male and female reconstructed pathways were parsed according to MAGs present in the sham or DCM groups and are available in Supplementary Table S8. Regarding central metabolism reactions, we reviewed all pyruvate associated pathways across MAGs present in the sham or DCM groups, across both sexes. In female MAGs, we evidenced that the pyruvate fermentation to propanoate and pyruvate decarboxilation to acetyl-CoA reactions were prevalent in DCM-associated MAGs and nearly absent in sham-associated MAGs (Figure 7E). The latter observations can also be applied across male MAGs (Figure 7F). The reactions involved in this process are summarized in Supplementary Table S9. The reaction in which pyruvate ferment to propanoate is complex and involve a fumarase, a fumarate reductase, a propionyl-CoA:succinate CoA transferase, a methylmalonyl-CoA carboxyltransferase and a malate dehydrogenase while the reactions in which pyruvate decarboxile to acetyl-CoA involve the presence of a pyruvate dehydrogenase NADP^+^ (Supplementary Table S9). Conversely, the conversion of pyruvate to butanoate involve three enzymes including a acetyl-CoA-acetyltransferase PaaJ and a Butyrate kinase (Buk). The latter reaction is frequently absent in MAGs present in DCM-induced dysbiosis across both sexes (Figures 7E,F and Supplementary Table S9). Since bacteria can ferment pyruvate down into butyrate via Butyrate kinase activity (Sievers et al., 2019) (diminished in DCM) and shuttle pyruvate into lactate through pyruvate dehydrogenase enzyme (Esquivel-Elizondo et al., 2017) (augmented in DCM), this imply that in the transition from sham to DCM-induced dysbiosis, the pool of butyrate among other metabolites in the microbiome such as propionate and acetate (Louis and Flint, 2017) could largely vary in DCM-induced dysbiosis.

### Butyrate production is halted in MAGs present in DCM-induced dysbiosis

**Figure 8:**
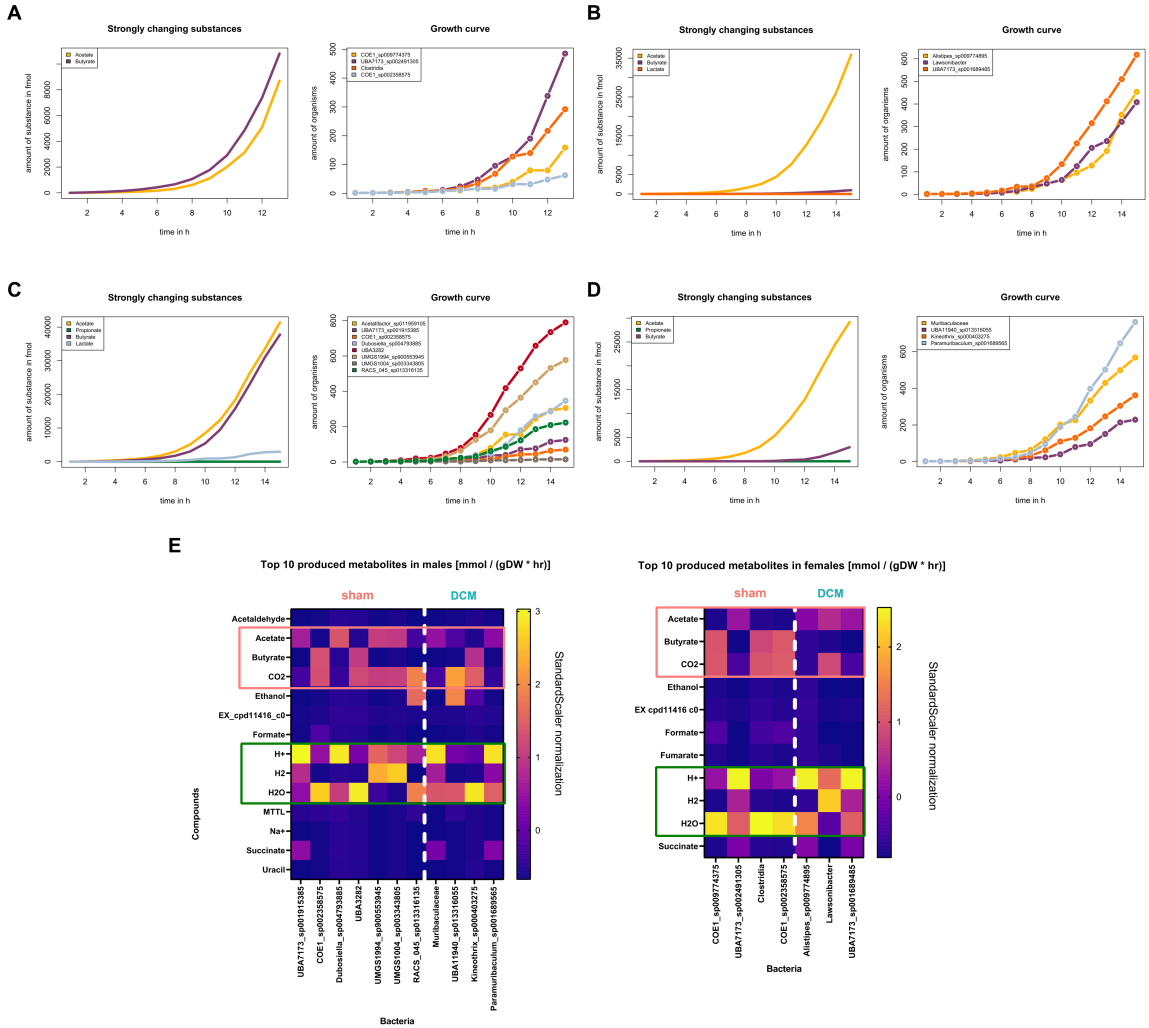
Butyrate production is halted in MAGs present in DCM-induced dysbiosis. **(A)** (Left) Female sham community simulation plot of strongly changed substances, modeled with gapseq gap-filling algorithm. The production of acetate and butyrate in femtomoles was plotted across simulation steps, denoted as time in hours (*n* = 14) (Right). Growth curves of female MAGs associated with sham condition across simulation steps, denoted as time in hours (*n* = 14). Each MAG was colored with a distinctive color. **(B)** Same as **(A)** for female DCM community simulation. In the graph at left, only acetate figure as a strongly changed substance. **(C)** Same as **(A)** for male sham community simulation. In the graph at left, acetate and butyrate figures as strongly changed substances. **(D)** Same as **(B)** for male DCM community simulation. In the graph at left, only acetate figure as a strongly changed substance. **(E)** Top 10 metabolites produced, measured in millimoles per gram dry weight per hour [mmol/(gDW*hr)], identified across males and females MAGs. The data is presented in two graphs, with a vertical line dividing sham and DCM associated MAGs in both sexes. In both males and females, the production of acetate is inversely proportional to the production of butyrate.

Based on the metabolic reconstruction across sham and DCM-associated MAGs in both sexes, we interrogated whether acetate, propionate and butyrate as fermentation products vary their concentrations between sham and DCM-induced dysbiosis conditions, respectively. This task is complicated in terms of predicting metabolite influx and efflux based solely of bioinformatic gene predictions, due complex metabolic networks and numerous biochemical transformations occurring in a microbiome community. Using gapseq modeling with a gap-filling approach, we simulated at community level the production of the mentioned metabolites using minimal medium composed of glucose and acetate. Across female MAGs present in sham condition, a steady increase of butyrate and acetate concentration was registered along the simulation’s steps with a marked growth increase of *UBA7173 sp002491305* (*Muribaculaceae* family) and *Clostridia sensu stricto* MAGs (Figure 8A). Conversely, when MAGs associated with DCM-induced dysbiosis were modeled under the same conditions, only acetate was a strongly changed substance with marked growth increase of all MAGs (Figure 8B). Same observations apply in the case of male MAGs modeling. Across male MAGs present in sham condition, both acetate and butyrate metabolites sharply increase along with marked growth of UBA3282 and UMGS1004 sp003334805 MAGs (Figure 8C). Conversely, under DCM-induced dysbiosis, only acetate increased its concentration displaying a marked growth increase of *Paramuribaculum sp001689565* (Muribaculaceae family) and *Muribaculaceae* MAGs (Figure 8D). In these simulations, butyrate metabolite production markedly increases when sham-associated MAGs are present while its production is nearly absent during DCM-induced dysbiosis, irrespective of the sex status. We interrogated the top 10 produced substances predicted by gapseq gap-filling modeling across male and female MAGs associated with sham (left) or DCM condition, respectively (Figure 8E). For better visualization, the predicted measurements in millimoles per gram dry weight per hour (mmol/(gDW*hr)) were normalized using a StandardScaler method in python. In this setting, across both males and females MAGs, the production of acetate is inversely proportional to the production of butyrate, and inversely proportional to the production of CO_2_ in less extent. This observation also supports that acetate can be a substrate for butyrate production, since as it has been described that butyrate-producing bacteria found in the human colon can be net users of acetate (Duncan et al., 2004). Overall, our metabolic simulations support the sustained production of butyrate in sham condition but in less extent in DCM condition, with the concomitant use of acetate, not produced by bacteria that synthetize butyrate in the mouse colon.

## Discussion

In this study through a combination of 16S rRNA sequencing and metagenomic approaches, we demonstrated for the first time that dysbiosis is present in the gut microbiome in the DCM mouse model, is sexually dimorphic, and leads to a predicted reduction in butyrate production. To our knowledge, this is the first study characterizing abundance and function of gut microbiome during the progression of DCM. Furthermore, we observed dynamic changes in T cells in females at the systemic and lamina propria level, and monocytes in the spleen in males.

Bidirectional gut-brain communication regulate homeostatic functions in our body, including immune regulation (Mayer et al., 2022). Innate and adaptive immune responses have been studied in the upper and lower GI tract, identifying regional immune compartmentalization of CD4^+^ and CD8^+^ T cells along the GI track (Mowat and Agace, 2014; Barreto de Alburquerque et al., 2022). The majority of immune processes take place in the mucosa, which is shaped by the epithelium, the lamina propria, and the muscularis mucosa. Among them, the lamina propria contains cells from the innate and adaptive immune system, and it is considered one of the main reservoirs for T cells in the body (Mowat and Agace, 2014). In our study, no differences were observed in the colon length, neither gastrointestinal function or constipation between sham and DCM groups. However, the frequency of Tregs in the female lamina propria was increased compared with the sham group. In mice, the number of Tregs in the colon is the highest along the length of the intestine. These cells produce large amounts of cytokines IL-10 and IL-17, and have been suggested to be implicated in the control of autoimmunity and inflammation (Voo et al., 2009). Specifically, translocation of Th17 cells from the gut to the brain has been involved in the development of inflammation during stroke (Singh et al., 2016). In humans the colon hosts the majority of microbial community in the body (Sender et al., 2016). Furthermore, at the systemic level, we also observed increased frequency of T cells, specifically the CD4^+^ T subset in females, confirming previously reported findings in the DCM mouse model (Ulndreaj et al., 2022). Of note, T cells have been suggested to play a key role during development of other pathologies, such as multiple sclerosis, amyotrophic lateral sclerosis (ALS) or aging (Broux et al., 2012; Bronge et al., 2022; Yazdani et al., 2022). A recent study has indicated that different CD4^+^ T subsets can act as predictor of disease progression following ALS (Yazdani et al., 2022). It has been suggested that colonic Tregs can be generated through contact with the gut microbiota (Atarashi et al., 2011; Lathrop et al., 2011; Nagashima et al., 2023). For example *Bifidobacterium, Lactobacillus, Bacteroides* can influence Treg development (Tan et al., 2022). Furthermore, germ free mice have a reduced number of colonic Tregs compared to specific-pathogen-free mice (Atarashi et al., 2011). Another potential mechanism is related to SCFAs, that can promote Foxp3 gene expression and Treg differentiation (Furusawa et al., 2013; Park et al., 2015). Thus suggesting that a higher number of Tregs might be associated with eubiosis and that a deeper understanding of the role of T cells during DCM is needed. Previous studies have suggested a crosstalk between the immune system and the microbiome. In mice the short chain fatty acid (SCFA) propionate has shown to regulate colonic Tregs size and function, promoting colonic homeostasis (Smith et al., 2013). Along the same line, *ex-vivo* studies have shown that bacterial extracts from microbiota of patients with non-alcoholic fatty liver disease elicited expansion of Tregs and attenuation of CD8^+^ T cells, monocytes and B cells expansion on peripheral blood mononuclear cells (Behary et al., 2021).

Here, using 16S rRNA sequencing and metagenomic approaches, we observed that DCM-induced dysbiosis in male was more pronounced that in the females. Along this line, a recent study has shown that changes in microbiota composition and metabolites can influence sex differences in circadian rhythms genes related to the immune system (Munyoki et al., 2023). Thus, it is tempting to suggest that gut dysbiosis might play a role here. This goes in agreement with human reports, where the prevalence of DCM patients was found to be greater in males than females (although GI comorbidities are more frequent in female patients) (Nouri et al., 2020). In females, under DCM-induced dysbiosis, we observed an increased abundance of several Gram-negative bacterial members from the order *Bacteroidales*, predominantly belonging to the *Muribaculaceae* family and in less extent to the *Rikenellaceae* family (genus Alistipes), respectively. We also observed in less extent the increase of the Gram-positive *Oscillospiraceae* family (genus Lawsonibacter). Later on, when symptomatology is fully developed there is an increased abundance of *Lachnospiracea* NK4A136 genus in the DCM group. In males during DCM-induced dysbiosis, we also detected a marked increase in abundance of several members of the mentioned *Muribaculaceae* family, accompanied in less extent with an increase of the Gram positive Kineothrix sp000403275 MAG (*Lachnospiraceae* family, order Lachnospirales). Similarly as in mice, in male SCI patients, the abundance of the Gram-negative commensal bacteria *Bacteroides* (order Bacteroidales) has been shown to be higher compared with healthy controls (Zhang et al., 2018), whereas in both, females and male patients, a reduction in members of the families *Lachnospiraceae,* as also seen in this study with the DCM mice model, has been reported (Gungor et al., 2016). Along this line, SCFA production, mainly butyrate, has been associated with various genera of the *Lachnospiraceae* family (Meehan and Beiko, 2014). Butyrate has been shown to contribute to maintain the gastrointestinal barrier and decrease inflammation (Mattace Raso et al., 2013; Ríos-Covián et al., 2016). Therefore, suggesting that a reduction in butyrate-producing bacteria can contribute to reduce integrity of the intestinal barrier and increased microbial translocation. Previous evidence in animal models of traumatic SCI, has shown changes in the abundance of *Bacteroidales*, *Clostridiales*, and *Bifidobacteriales* (Kigerl et al., 2016; O'Connor et al., 2018). Furthermore, the abundance of *Bacteroides* and *Lachnospiracea* have been directly correlated with SCI severity (Bazzocchi et al., 2021). The same study, reported that SCI patients could also be stratified by lesion level based on microbiota composition (Bazzocchi et al., 2021). One alternative to restore microbiome composition is via fecal microbiota transplants (FMT) from healthy donors. A study carried out in mice with traumatic SCI who were transplanted with fecal microbiota from healthy mice, demonstrated that this technique improved the locomotor recovery of injured mice, as well as other parameters (i.e., metabolic profile, inflammation, integrity of the intestinal barrier, and gastrointestinal motility) by ameliorating gut dysbiosis (Jing et al., 2021). Thus, it is tempting to suggest that prevention of DCM-induced dysbiosis would slow down disease progression by ameliorating locomotor symptomatology.

In other scenarios, dysbiosis has been correlated with a drastic change of bacterial populations and with the consequently production of metabolites. As example, in a mouse model of sciatic nerve crush the production of the metabolite indole-3 propionate (IPA) by gut *Clostridiales* order *(Clostridium sporogenes)* has been shown to accelerate axonal regeneration by promoting communication between neutrophils and dorsal root ganglion (DRG). IPA is a product of bacterial metabolism of tryptophan in the gut (Serger et al., 2022). Additionally, in a model used to enhance thermogenic and mitochondrial activity in adipose tissue, by using mice cold-exposed as well as transplantation of cold-exposed mice into germ-free mice, has also reported changes in microbiota composition. Specifically, this study observed an increase of the family *Lachnospiraceae* and a decrease in *Muribaculaceae* and *Oscillospiraceae families,* along with an augment of butyrate, propionate, succinate, and lactate production (Kieser et al., 2022).

In line with the latter, SCFAs (i.e., butyrate, acetate, and propionate) are found at higher concentrations in the colon. They are generated by anaerobic digestion of oligosaccharides by *Firmicutes* and *Bacteroidetes* (Mowat and Agace, 2014). SCFAs have also been involved in immune cell function (i.e., microglial cells maturation and phagocytosis during neurodenegeneration) (Erny et al., 2021). In this study, we predicted in both female and males, that MAGs associated with DCM-induced dysbiosis tend to lack genes such as *butyrate kinase* (Buk), involved in the fermentation of pyruvate toward butyrate. Conversely, the latter MAGs present the *lactate dehydrogenase* enzyme that converts pyruvate into lactate. It was hypothetized that in the transition from sham to DCM-induced dysbiosis, the pool of butyrate, propionate and acetate greatly vary in DCM-induced dysbiosis. Gapseq-simulation at the community level of these MAGs confirmed the lack of butyrate production under DCM-induced dysbiosis in both sexes, along with the increase of MAGs belonging to *Muribaculaceae* family. This simulation correlates with the lack of butyrate producing bacteria as a hallmark of bacterial dysbiosis, since these bacteria are often depleted in the diseased gut microbiota and its restoration improve several inflammatory diseases, as previously reported (Zhang Q. et al., 2016; Geirnaert et al., 2017; Parada Venegas et al., 2019).

Additionally, we predicted increase in LPS signaling in male DCM-associated MAGs. A recent study has shown that gut dysbiosis contributes to mastitis by increasing bacterial translocation and systemic inflammation. Specifically, the authors reported a reduction in butyrate-producing bacteria and increasing LPS levels in serum. Treatment with fecal microbiota transplant supplemented with *Roseburia* attenuated gut-dysbiosis induced mastitis by increasing production of butyrate, which was associated with attenuation of bacterial translocation and inflammation, as well as gut barrier repair (Zhao et al., 2022). Furthermore, following traumatic SCI in humans, it has also been reported a reduction in butyrate-producing bacteria (Gungor et al., 2016). Butyrate has also been shown to contribute to down-regulate inflammation, by controlling gene expression in the gut-associated immune system (Magnusson et al., 2020), and regulation of Treg/Th17 differentiation and activation (Zhang M. et al., 2016). Based on this evidence, the downregulation of butyrate through the diminished abundance of butyrate-producing bacteria along with an increase of bacteria that produce LPS is a common consequence of gut dysbiosis, also evidenced in this study.

Recently, it has been demonstrated that the metabolic interaction between the host and the gut microbiome is very complex, involving both the microbiome and the genetics of the host. A recent study performing metabolome-genome-wide association analysis identified that 54.6% of blood metabolites are positively associated with the gut microbiome composition. To make things more complex, the same study identified that metabolites derived from gut microbial precursors can also be influenced by host genetic variations (Diener et al., 2022). Thus, for some blood metabolites their levels are a combination of gut microbiome and genetic. In our study, some of the metabolites highly expressed in the DCM group in males [i.e., 3-(4-hydroxyphenyl) pyruvate biosynthesis, pyruvate fermentation to acetate II or butanoate] have been associated with an hybrid component, host genetic and gut microbiome, or solely with the microbiome (Diener et al., 2022).

Our study has certain limitations. Although mouse is the most used model for studying the microbiota, a recently comprehensive mouse microbiota genome collection has shown that human and mouse gut microbiome overlap 80% at the family level and 13% at the species level (Kieser et al., 2022). Thus, future studies should aim to characterize human DCM microbiome using shotgun to include uncultured microorganisms and viruses in their analysis. Furthermore, future studies will need to validate the impact of the inference molecules of interest presented in this manuscript (i.e., LPS and butyrate) using targeted metabolomics of fecal samples.

Importantly, the present study propose that certain bacterial members from the *Muribaculaceae* family are strongly associated with DCM-induced dysbiosis while several members of the *Lachonospiraceae* family greatly diminished its levels in this condition. Through metabolic modeling at the bacterial community level, it is expected that Butyrate is strongly diminished during DCM-induced dysbiosis, a recurrent phenotype in gut-associated dysbiosis. Finally, we propose a molecular mechanisms that explain the absence of butyrate production in this context.

## Conclusion

This study reports, for the first time, that DCM induces sexually dimorphic gut dysbiosis. We propose that bacterial members of the *Muribaculaceae* family are candidates of DCM-induced dysbiosis in mice. DCM-induced dysbiosis leads to a reduction of the abundance of both butyrate and butyrate-producing bacteria that is predicted to diminish dramatically the concentration levels during dysbiosis. The latter result is supported through metagenomic identification of candidate genes and community-based metabolic simulations. Since, DCM-induced dysbiosis is stronger in males than females, our results provide new evidence to better understand the greater prevalence of DCM in males versus females and to further support the development of personalized medicine approaches to this common form of non-traumatic SCI.

## Data availability statement

The datasets presented in this study can be found in online repositories. The names of the repository/repositories and accession number(s) can be found in the article/Supplementary material.

## Ethics statement

The animal study was approved by the Animal Use Committee (AUC) of the Universidad Católica de la Santísima Concepción, Concepción, Chile. The study was conducted in accordance with the local legislation and institutional requirements.

## Author contributions

PMV conceived the study and the experimental design and performed surgical procedures and flow cytometry experiments. CF performed bioinformatic analysis. AÁ and ER-F performed immunohistochemistry and reconstruction. All authors contributed to writing the manuscript.

## Figures and Tables

**Table 1:** 

ASVs	Amplicon Sequence Variants
ALS	Amyotrophic lateral sclerosis
ANOVA	Analysis of variance
AUC	Animal Use Committee
Buk	Butyrate kinase
CNS	Central Nervous System
COGs	Clusters of Orthologus Groups
DAPI	4′,6-diamidino-2-phenylindole
DCM	Degenerative Cervical Myelopathy
DRG	Dorsal root ganglion
FDR	False Discovery Rate
GI	Gastrointestinal
GALT	Gut-associated lymphoid tissues
IPA	Indole-3 propionate
iTOL	Interactive Tree of Life
LPS	Lipopolysaccharide
MAGs	Metagenomes-assembled genomes
NMDS	Non-metric Multi-dimensional Scaling
ORFs	Open reading frames
PFA	Paraformaldehyde
PBS	Phosphate buffered saline
PRPP	Phosphoribosyl pyrophosphate
RT	Room temperature
SEM	Standard error of the mean
SCI	Spinal cord injury
Tregs	Regulatory T cells

## Footnotes

^1^https://github.com/tseemann/barrnap

^2^https://github.com/cfarkas/annotate_my_genomes/wiki#1-obtaining-parsed_resultstab-file

^3^https://github.com/christophergandrud/networkD3

^4^https://pandas.pydata.org/
