# Supplementary material for: Degenerative Cervical Myelopathy induces sex-specific dysbiosis in mice
Source: Front Microbiol. 2023 Oct 20;14:1229783. doi: 10.3389/fmicb.2023.1229783 (PMC10623434; doi:10.3389/fmicb.2023.1229783)
Supplement: Supplementary file 10 [file Presentation_1.pdf]

Supplementary Material

Degenerative Cervical Myelopathy induces sex-specific dysbiosis in mice

Carlos Farkas[1], Eduardo Retamal-Fredes[2], Ariel Ávila[2] , Michael G. Fehlings[3,4], Pia M. Vidal[5,*]

[1] Biomedical Science Research laboratory, Basic Sciences Department, Faculty of Medicine, Universidad Católica de la Santísima Concepción, Concepción, Chile .

[2] Developmental Neurobiology Unit, Biomedical Science Research laboratory, Basic Sciences Department, Faculty of Medicine, Universidad Católica de la Santísima Concepción, Concepción, Chile.

[3] Department of Genetics and Development, Krembil Research Institute, University Health Network, Toronto, ON, Canada.

[4] Spinal Program, University Health Network, Toronto Western Hospital, Toronto, ON, Canada.

[5] Neuroimmunology and Regeneration of the Central Nervous System Unit, Biomedical Science Research laboratory, Basic Sciences Department, Faculty of Medicine, Universidad Católica de la Santísima Concepción, Concepción, Chile.

*** Correspondence:** Corresponding Author: Pía M. Vidal, Ph.D: pvidal@ucsc.cl

Supplementary Figure 1: Error rate plots of all-to-all nucleotide comparisons across male (upper panel) samples and female (lower panel) samples. The error frequency (log 10) in the Y-axis was plotted against the consensus quality score in the X-axis.

Supplementary Table 1: Original paired-end sequencing labels of the 16S rRNA sequencing datasets and the assigned name in this study. The label contains the following letters as label: Gender (M or F), Treatment (T1, T2, T3, C1, C2 and C3, respectively) and Block (sample number).

Supplementary Table 2: DADA2-produced table and taxonomy species assigned to each ASV, outputs of ampliseq nf/core nextflow pipeline.

Supplementary Table 3: Differentially abundant ASVs across female and male samples, respectively with the corresponding Z-score of abundance, outputs obtained with edgeRun R package. The input used for these calculations was the DADA2 abundance output table (in csv format).

Supplementary Table 4: Matched MAGs from iMGMC database with >95% identity against male and female ASVs, respectively. The taxonomy was resolved using GTDB database.

Supplementary Table 5: Pangenomics analysis of female and male MAGs. Gene presence-absence tables were included across female and male MAGs, including common sets of genes shared by all MAGs identified in this study.

Supplementary Table 6: Pathway annotation and enriched metabolic pathways across female MAGs. A pathway with an adjusted q-value over 0.05 was considered significant.

Supplementary Table 7: Pathway annotation and enriched metabolic pathways across male MAGs. A pathway with an adjusted q-value over 0.05 was considered significant.

Supplementary Table 8: gapseq pathway annotation across female and male MAGs, respectively. The presence of a given pathway/reaction was denoted with ones and the absence with zeros.

Supplementary Table 9: Pyruvate-related reactions and genes identified across sham or DCM MAGs in males and females, respectively using gapseq.
