# Supplementary figures and images for: Degenerative Cervical Myelopathy induces sex-specific dysbiosis in mice
Source: Front Microbiol. 2023 Oct 20;14:1229783. doi: 10.3389/fmicb.2023.1229783 (PMC10623434; doi:10.3389/fmicb.2023.1229783)

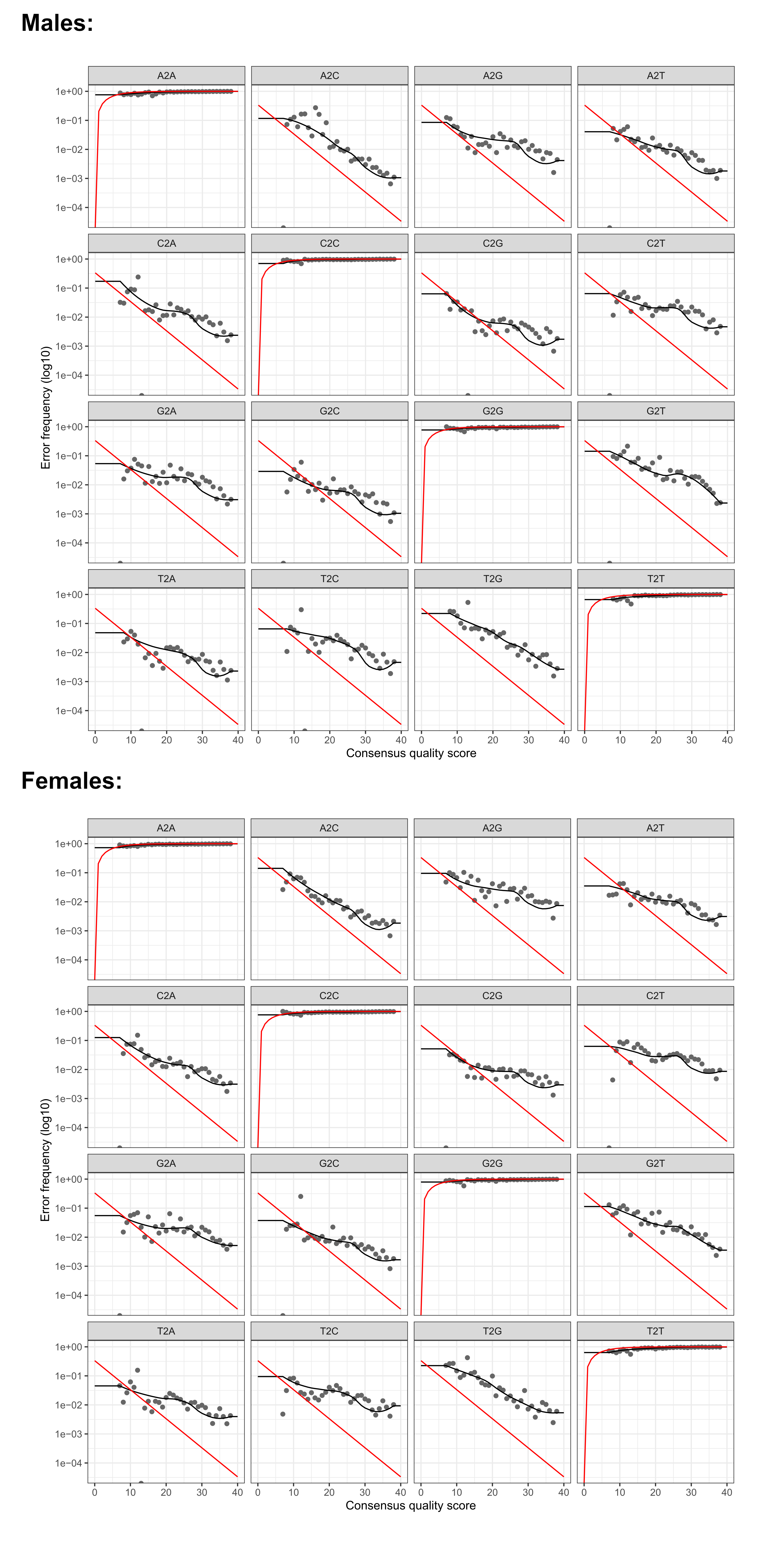

Supplement: Supplementary file 11 [file Image_1.TIF]
